# A New Clinical Classification Method for Gynecomastia Based on Chest Appearance and MR/CT Images

**DOI:** 10.1007/s00266-025-05196-x

**Published:** 2025-10-29

**Authors:** Jiawei Gu, Haiyang Hu, Jianghui Ying, Qianhui Xu, Lu Lu, Xiaoyan Yu, Ni Zhuang, Jie Zhou, Hua Jiang, Hui Wang

**Affiliations:** 1 Department of Plastic Surgery, Shanghai East Hospital, School of Medicine, Tongji University, 150 Jimo Road, Shanghai, 200120 China; 2 Department of Radiology, Shanghai East Hospital, School of Medicine, Tongji University, 150 Jimo Road, Shanghai, 200120 China

**Keywords:** Gynecomastia, Clinical classification, Chest appearance, MR/CT images

## Abstract

**Background:**

Classification theories for gynecomastia often conflate breast ptosis degree with subcutaneous soft tissue composition, leading to further confusion in determining appropriate surgical regions and techniques. To address this, we propose a new clinical classification method for gynecomastia.

**Methods:**

We first divided each side of male chest into six distinct regions, then separated the concept of grade and type in gynecomastia classification. Five grades were defined based on chest appearance and breast ptosis (skin redundancy). Grade 1–4 Patients underwent surgery in region 1–4 accordingly, with region 5–6 addressed if necessary. Grade 5 patients received treatment in all six regions. Two types with six subtypes were defined based on the proportion and distribution of subcutaneous glandular tissue shown in MR/CT images. Type 1 (A-C) patients typically required liposuction alone, with pull-through added if necessary, while Type 2 (A/B) patients generally required additional pull-through. For Type 2C patients, mammotome-assisted resection was used to remove dense, enlarged glands.

**Results:**

Two hundred and ninety-seven gynecomastia patients were included in this study. There were 32, 183, 45, 12, and 5 patients in grade 1–5, respectively, along with 20 patients with asymmetric gynecomastia. A total of 132 patients underwent preoperative MR/CT imaging, with 85 classified as Type 1, 39 as Type 2, and 8 as asymmetric cases.

**Conclusion:**

We propose a new gynecomastia classification method based on chest appearance and MR/CT images, distinguishing grade from type. Grade determines the surgical region, while type guides the choice of surgical technique. This approach is objective and clinically meaningful.

**Level of Evidence IV:**

This journal requires that authors assign a level of evidence to each article. For a full description of these Evidence-Based Medicine ratings, please refer to the Table of Contents or the online Instructions to Authors www.springer.com/00266.

## Introduction

Gynecomastia usually refers to the appearance of male breast showing the characteristics of female breast. Most patients with gynecomastia exhibit varying degrees of benign mammary gland proliferation beneath the nipple-areola complex (NAC). However, some patients show only an increase in subcutaneous fat tissue, without significant glandular proliferation or enlargement, which is typically referred to as pseudogynecomastia. Physiological gynecomastia most commonly occurs during three peak periods: infancy, adolescence, and after the age of 50 [1]. While pathological gynecomastia can occur in cases with a history of specific drug use, tumors, chronic kidney disease, Klinefelter syndrome, and other underlying conditions [2]. For most adult patients with gynecomastia, the proliferated mammary gland tissue does not regress spontaneously and can only be removed through surgical intervention. Patients often seek medical consultation for psychological reasons, hoping to improve the appearance of their chest and alleviate social embarrassment [3].

In 1946, Webster first proposed a clinical classification system and corresponding surgical approach for gynecomastia. He identified three types of gynecomastia based on the ratio of connective tissue around the mammary glands to fatty tissue. For all three types, the primary surgical approach involved excising excess glandular tissue through an incision below the areola [4]. Later, in 1973, Simon introduced the widely recognized Simon classification system. He classified gynecomastia into three types based on the degree of breast enlargement and the presence of skin redundancy [5]. With the advancement of liposuction techniques, Rohrich proposed a classification system in 2003 based on liposuction [6]. While this system provides clearer and more quantifiable criteria for classification, it still relies on subjective judgment and surgeons’ clinical experience. In recent years, the advancement of various surgical techniques, including vacuum-assisted liposuction, pull-through (PT) technique, mammotome-assisted resection, and endoscopic-assisted resection, has significantly expanded the surgical treatment options for gynecomastia [7–9]. Numerous scholars have proposed different clinical classification systems for gynecomastia from various perspectives. Key factors commonly considered in these classification systems include the composition ratio of adipose tissue and glandular tissue, the presence of the inframammary fold (IMF), and the degree of skin redundancy. These factors play a crucial role in assessing the severity of gynecomastia and guiding treatment decisions, particularly in selecting the most appropriate surgical approach. However, most classification methods primarily rely on surgeons’ physical examination and clinical experience, which inherently have limitations. Consequently, these systems may not comprehensively capture the diverse characteristics of individual patients, thereby restricting their effectiveness in guiding surgical decision-making [10–13].

Developing a more effective clinical classification system for gynecomastia is essential to optimizing surgical approach selection, minimizing surgical trauma, accelerating postoperative recovery, and enhancing patient outcomes. In this study, we introduce a more objective and quantifiable classification method based on chest appearance and preoperative MR/CT imaging of gynecomastia patients.

## Methods

### Patient Source and Ethical Approval

This study collected data from gynecomastia patients who underwent surgical treatment at the Department of Plastic Surgery, East Hospital, Tongji University, between January 2021 and December 2024. The inclusion criteria were as follows: (1) patients aged 18 to 60 years; (2) no prior surgical history for gynecomastia; and (3) no soft tissue masses or lesions in the chest subcutaneous area. All medical procedures and examinations were thoroughly explained to the patients, and informed consent was obtained prior to any interventions. This study was approved by the Ethics Committee of East Hospital, Tongji University.

### Preoperative Imaging and Image Processing

For patients undergoing general anesthesia, a preoperative chest CT scan was performed to exclude any pulmonary contraindications. For those receiving local anesthesia who consented to preoperative MR, scans were performed accordingly. In cases where patients decline the MR, an ultrasound examination was conducted to rule out subcutaneous lesions. 3D Slicer software (Version 5.6.2) was used to reconstruct the glandular tissue in three dimensions, enabling a detailed analysis of its distribution pattern and volume.

### Preoperative Preparation, Postoperative Treatment, and Follow-Up Schedule

All patients underwent routine physical examinations perioperatively. The size of NAC and other chest surface markers were recorded. Two senior plastic surgeons determined the surgical region and techniques based on patients’ condition. The grade of gynecomastia was assessed based on chest appearance to guide the surgical region, while the type of gynecomastia was evaluated using MR/CT imaging to guide the choice of surgical techniques. Postoperatively, compression garments were used to promote skin retraction and enhance contour shaping. Follow-up visits were scheduled at 1-day, 3-day, 7-day, 1-month, 3-month, 6-month, and 12-month post-surgery.

### Surgical Procedures

Liposuction: the tumescent solution was prepared with 1 mL of adrenaline, 20 mL of 2% lidocaine hydrochloride, and 1000 mL of saline solution. The patient was positioned supine with both upper limbs externally rotated at a 90° angle. A small 0.3 cm incision was made along the anterior axillary line, just 1–2 cm below IMF, where a 0.3 cm incision protector was then securely placed. The designated surgical area was infiltrated with the prepared tumescent solution. After 20 min, a 0.3 cm cannula was inserted through the incision protector into the subcutaneous fat layer to remove fat tissue from the surgical area.

Pull-through technique: After completing the basic liposuction, pull-through technique was operated if residual glandular tissue remained visible and affected breast appearance. A 1 to 1.5 cm incision was made or extended from the liposuction incision site. The remaining glandular tissue was carefully grasped and pulled out through the small incision using suitably sized clamps, and then dissected using scissors. A certain thickness of retroareolar tissue was preserved to prevent local skin necrosis and ensure smooth skin contour [14, 15].

Mammotome‐assisted resection: This surgical technique was recommended for patients with large, dense glandular tissue judged by preoperative imaging results. The cutting head was used to puncture the skin at the same incision site as mentioned above before liposuction. Under ultrasound guidance, the cutting head was then advanced into the interlayer between the mammary glandular tissue. The majority of the glandular tissue and part of the surrounding fat tissue were removed. Care should be taken to avoid excessive resection below the areola, as this may result in ischemic necrosis due to insufficient blood supply.

### Definition of Chest Region (Fig. 1)

**Fig. 1:**
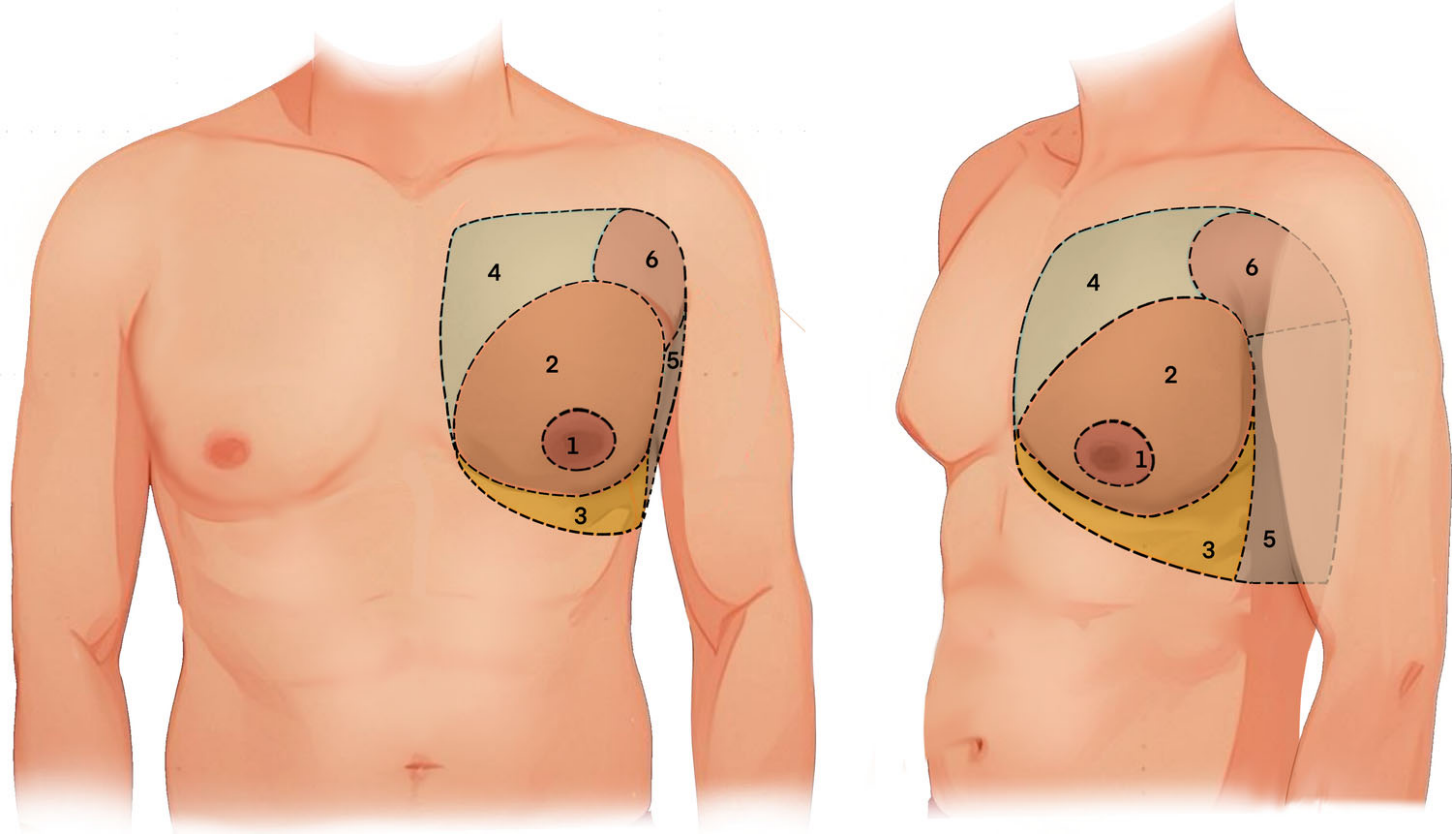
The anatomical illustration of male chest region in frontal (left) and oblique (right) view

Region 1: NAC region. The elliptical area extending 1.5 cm outward from the NAC boundary.

Region 2: Breast region. The protrusion breast area around region 1. The medial boundary is within the lateral sternal line. The lateral boundary is within the anterior axillary line. The superior boundary does not exceed the lower half of the pectoralis major muscle. The inferior boundary is defined by the IMF.

Region 3: IMF region. A crescent-shaped area below region 2. The medial boundary is within the lateral sternal line. The lateral boundary is within the anterior axillary line. The superior boundary is the IMF. The inferior boundary is 2 cm below the IMF.

Region 4: Upper chest region. The medial boundary is the within lateral sternal line. The lateral boundary is within the anterior axillary line. The superior boundary is the horizontal line of the sternal angle. The inferior boundary is adjacent to region 2.

Region 5: Lateral chest region. The region between the pectoralis major and latissimus dorsi muscles. The anterior boundary is within the anterior axillary line. The posterior boundary is within the posterior axillary line. The superior boundary is the lower edge of the axilla. The inferior boundary is defined by a line extending from the IMF toward the lateral and posterior direction.

Region 6: Accessory breast region. The upper outer quadrant region of the chest, near the axilla. In some patients, this area has a distinct boundary from region 1. The medial boundary is within the midline of clavicle. The lateral boundary extends beyond the anterior axillary line but within the posterior axillary line. The superior boundary is the horizontal line of the sternal angle. The inferior boundary is adjacent to region 1 and region 5.

### Grade of Gynecomastia and Corresponding Surgical Region (Table 1 and Fig. 2)

**Table 1:** Grade of gynecomastia and corresponding surgical region

Grade	Breast appearance	Surgical region
Grade 1	No IMF	Region 1 (+ 5/6)
Grade 2	IMF, without skin redundancy	Region 1 + 2 (+ 5/6)
Grade 3	IMF, with skin redundancy, NAC above IMF	Region 1 + 2 + 3 (+ 5/6)
Grade 4	IMF, NAC at the same height or 1cm below IMF	Region 1 + 2 + 3 + 4 (+ 5/6)
Grade 5	IMF, NAC more than 1 cm below IMF	Region 1 + 2 + 3 + 4 + 5 + 6

**Fig. 2:**
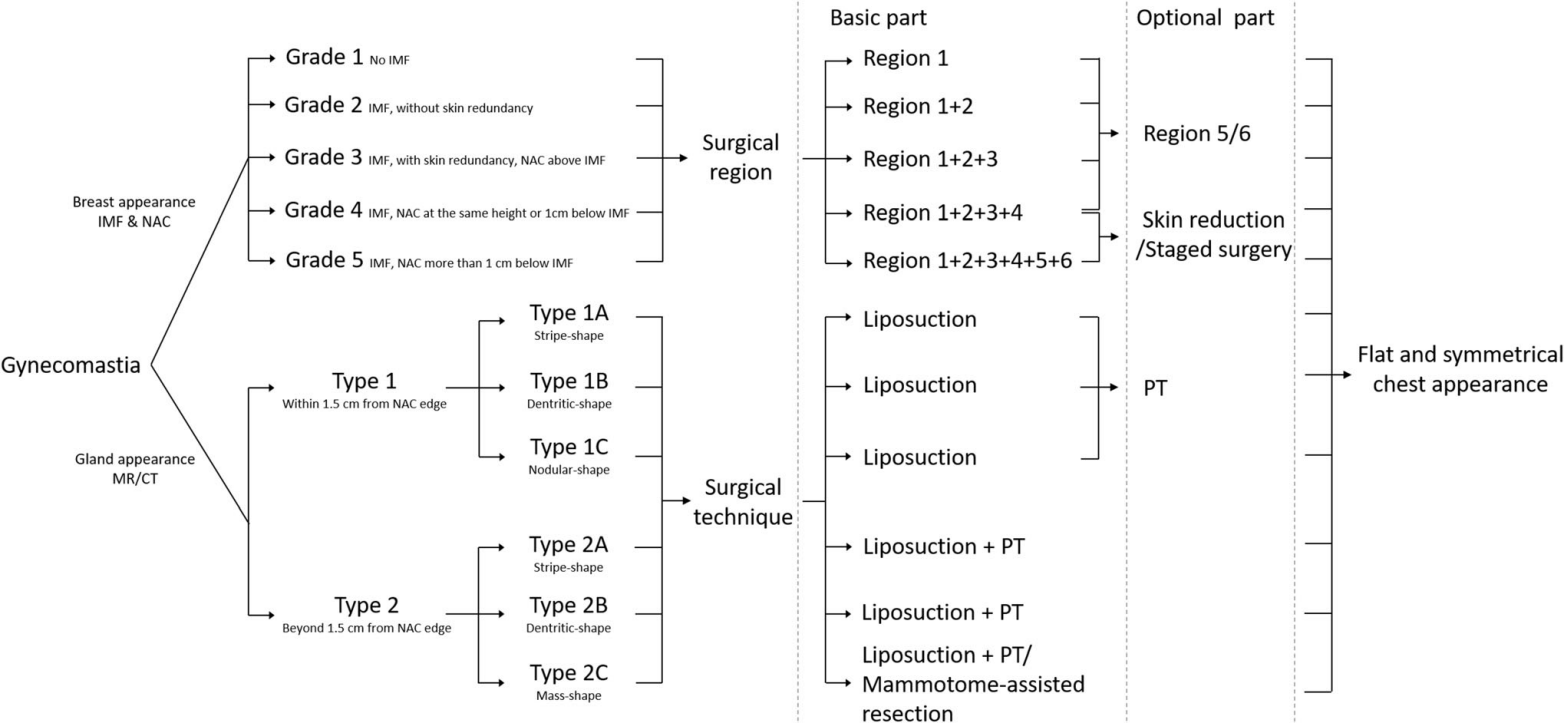
Illustration of classification algorithm

Grade 1: No IMF is observed. Only region 1 shows certain localized protrusion. Surgical procedures are focused on region 1.

Grade 2: IMF is observed. Region 1 and 2 show localized protrusion without skin redundancy. Surgical procedures are focused on region 1 and 2.

Grade 3: IMF is observed. Region 1 and 2 show localized protrusion, with skin redundancy. NAC is above the IMF. Surgical procedures are focused on region 1–3.

Grade 4: IMF is observed. The entire breast shows protrusion with skin redundancy. NAC is at the same height as or 1cm below IMF. Surgical procedures are focused on region 1–4. Subcutaneous tissue is released and dissected for skin reshaping. Strict bandaging and compression molding of the entire chest are required postoperatively.

Grade 5: IMF is observed. The entire breast shows protrusion with skin redundancy. NAC is more than 1 cm below IMF. Surgical procedures are focused on region 1–6. Subcutaneous tissue is released and dissected for skin reshaping. Strict bandaging and compression molding of the entire chest are required postoperatively. Some patients may consider staged surgical treatment for a better postoperative outcome. For patients accepting postoperative skin scars, excision of redundant skin may be performed.

Based on this, an additional evaluation of region 5 and 6 should be conducted for patients with grades 1–4. For those with evident protrusion in region 5 and 6, liposuction is recommended to remove subcutaneous fat. For region 5, care must be taken at the junction position between the lateral chest and lateral abdomen. For patients with developed musculature, attention should be given to reinforcing the posterior border of the pectoralis major and the anterior border of the latissimus dorsi. For region 6, it is crucial to avoid damaging the deep blood vessels and nerves. The skin in the axillary breast area is relatively loose, making postoperative bandaging and compression vital to reduce edema and promote recovery.

### Type of Gynecomastia and Corresponding Surgical Techniques (Table 2 and Fig. 2)

**Table 2:** Type of gynecomastia and corresponding surgical techniques

Type	Gland appearance in transverse plane	Surgical technique
Type 1	Within 1.5 cm from NAC edge	
1A	Stripe-shape	Liposuction (+ PT)
1B	Dentritic-shape	Liposuction (+ PT)
1C	Nodular-shape	Liposuction (+ PT)
Type 2	Beyond 1.5 cm from NAC edge	
2A	Stripe-shape	Liposuction + PT
2B	Dentritic-shape	Liposuction + PT
2C	Mass-shape	Liposuction + PT/Mammotome-assisted resection

Type 1: The gland tissue is within 1.5 cm from the edge of NAC. Based on MR/CT transverse plane images, it can be classified into stripe-shape (1A), dentritic-shape (1B), or nodular-shape (1C). Type 1 gynecomastia patients generally have a small volume of gland tissue that is localized near the NAC. Most cases can achieve satisfactory surgical outcomes with liposuction alone, although some patients may require additional PT excision to remove residual gland tissue, usually judged intraoperatively by surgeons.

Type 2: The gland tissue extends beyond 1.5 cm from the edge of NAC. Based on MR/CT transverse plane images, it can be further classified into stripe-shape (2A), dentritic-shape (2B), or mass-shape (2C). For type 2A and 2B, PT is usually necessary to remove the residual glandular tissue after liposuction. For type 2C, mammotome-assisted resection could first be used to remove the dense glandular tissue, followed by liposuction to address the surrounding fat tissue.

## Results

### Grade

**Fig. 3:**
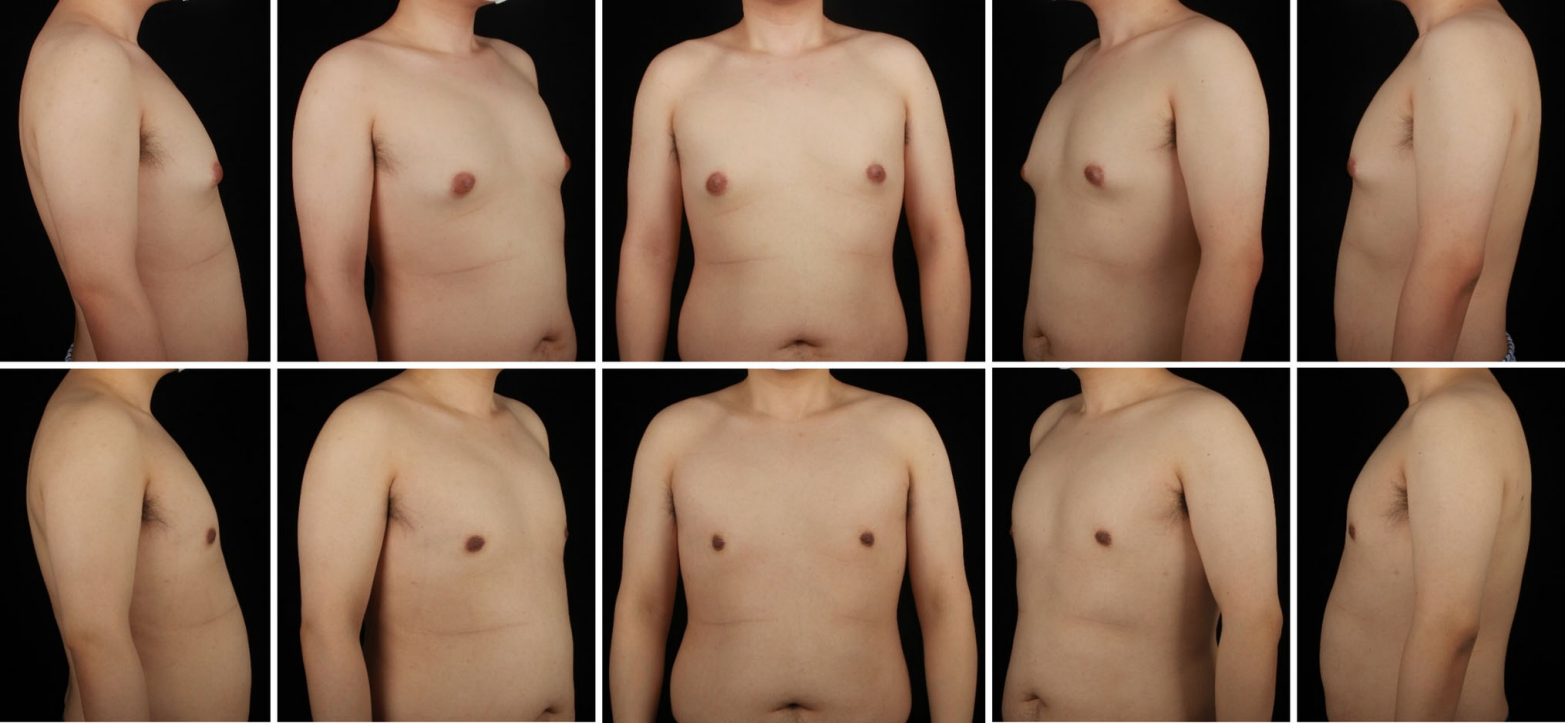
Grade 1 gynecomastia. Preoperative chest appearance (upper) and postoperative result two years later (lower) of a 28-year-old man with grade 1 gynecomastia

**Fig. 4:**
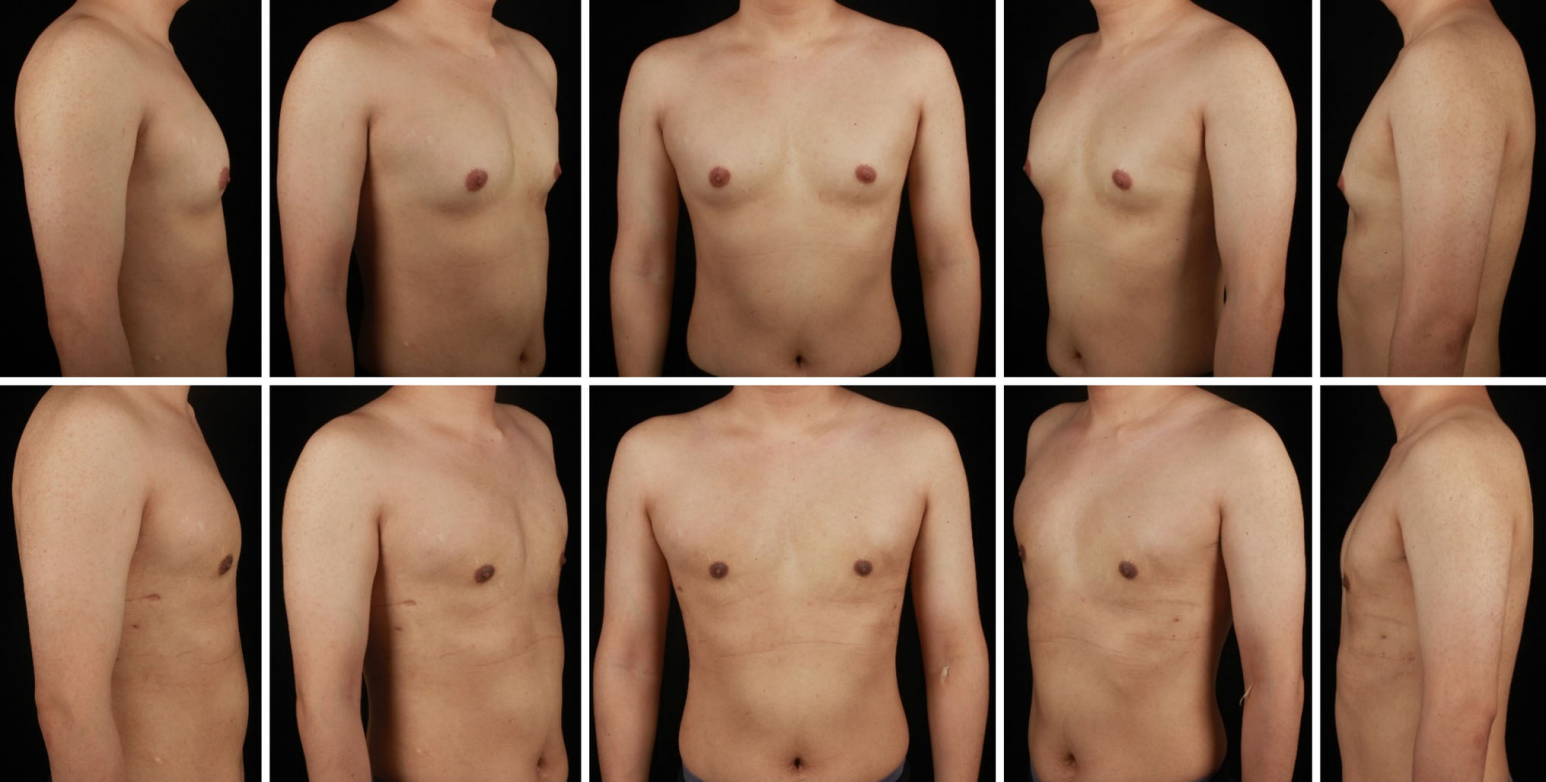
Grade 2 gynecomastia. Preoperative chest appearance (upper) and postoperative result half years later (lower) of a 27-year-old man with grade 2 gynecomastia

**Fig. 5:**
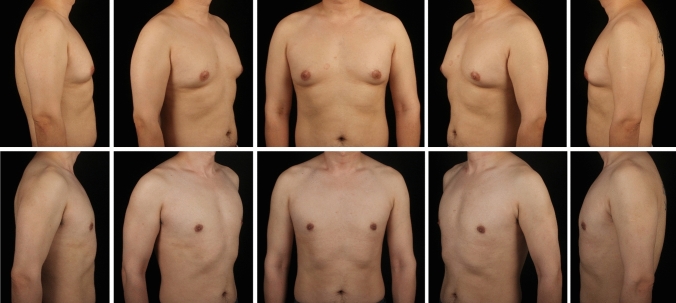
Grade 3 gynecomastia. Preoperative chest appearance (upper) and postoperative result two years later (lower) of a 38-year-old man with grade 3 gynecomastia

**Fig. 6:**
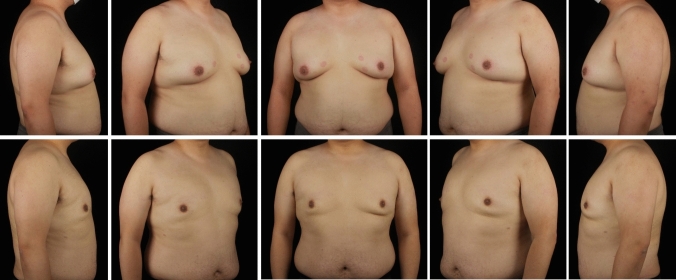
Grade 4 gynecomastia. Preoperative chest appearance (upper) and postoperative result one years later (lower) of a 35-year-old man with grade 4 gynecomastia

**Fig. 7:**
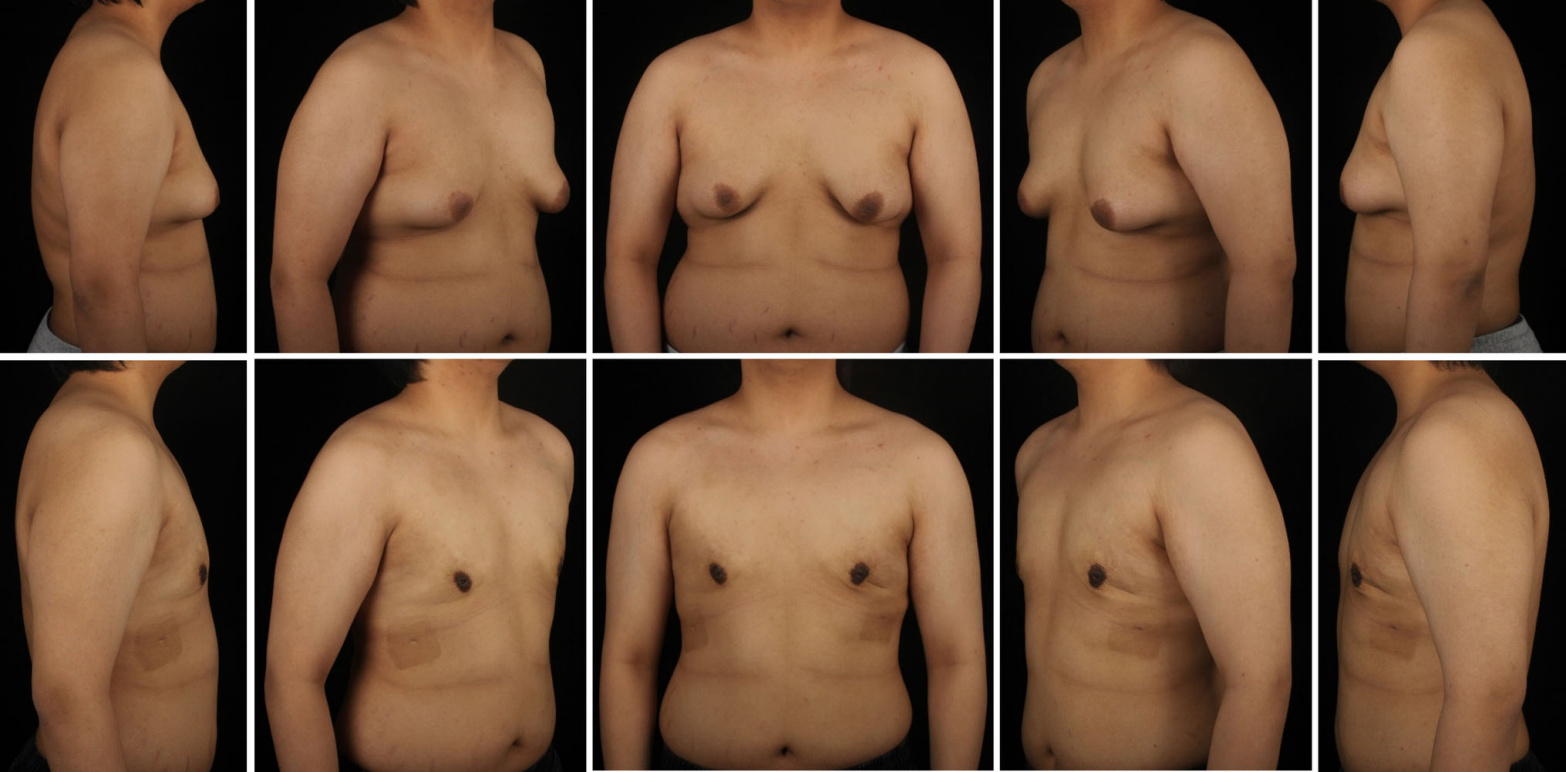
Grade 5 gynecomastia. Preoperative chest appearance (upper) and postoperative result seven months later (lower) of an 18-year-old man with grade 5 gynecomastia

A total of 297 gynecomastia patients were included in this study. The mean age was 29.9 (7.1) years, and the mean BMI was 25.59 (3.29). The grade of all included symmetrical gynecomastia patients is shown in Table 3. Grade 1 patients exhibit only localized protrusions of NAC, primarily seeking surgical intervention for psychological or social reasons. Some patients have specific esthetic demands for the appearance of the pectoralis major muscle, due to fitness needs. There is usually a slight local bulge within 1.5 cm of the NAC, which is more evident in the lateral position (Fig. 3). Most grade 1 patients do not need operation on region 5 or 6. Grade 2 is the most common type. The IMF is visible, though not as pronounced as in grade 3 patients (Fig. 4). While grade 3 patients show distinct skin redundancy and IMF (Fig. 5). The destruction of region 3 is important for skin retraction and remolding. For grade 4 patients, particularly in tubular breast cases, surgical treatment on region 1–4 is sufficient. However, most grade 4 patients exhibit a generally enlarged chest, necessitating surgical procedures on regions 5 and 6 as well (Fig. 6). This is same in Grade 5 patients (Fig. 7). For grade 4 and 5 patients, postoperative bandaging and compression are critical.

**Table 3:** Grade of symmetrical gynecomastia patients

Grade	Number of patients	Region 5	Region 6	Region 5 and 6	None region 5 or 6
Grade 1	32	1	3	0	28
Grade 2	183	11	61	21	90
Grade 3	45	5	12	17	11
Grade 4	12	0	0	11	1
Grade 5	5	0	0	5	0
Total	277	17	76	54	130

### Type

**Fig. 8:**
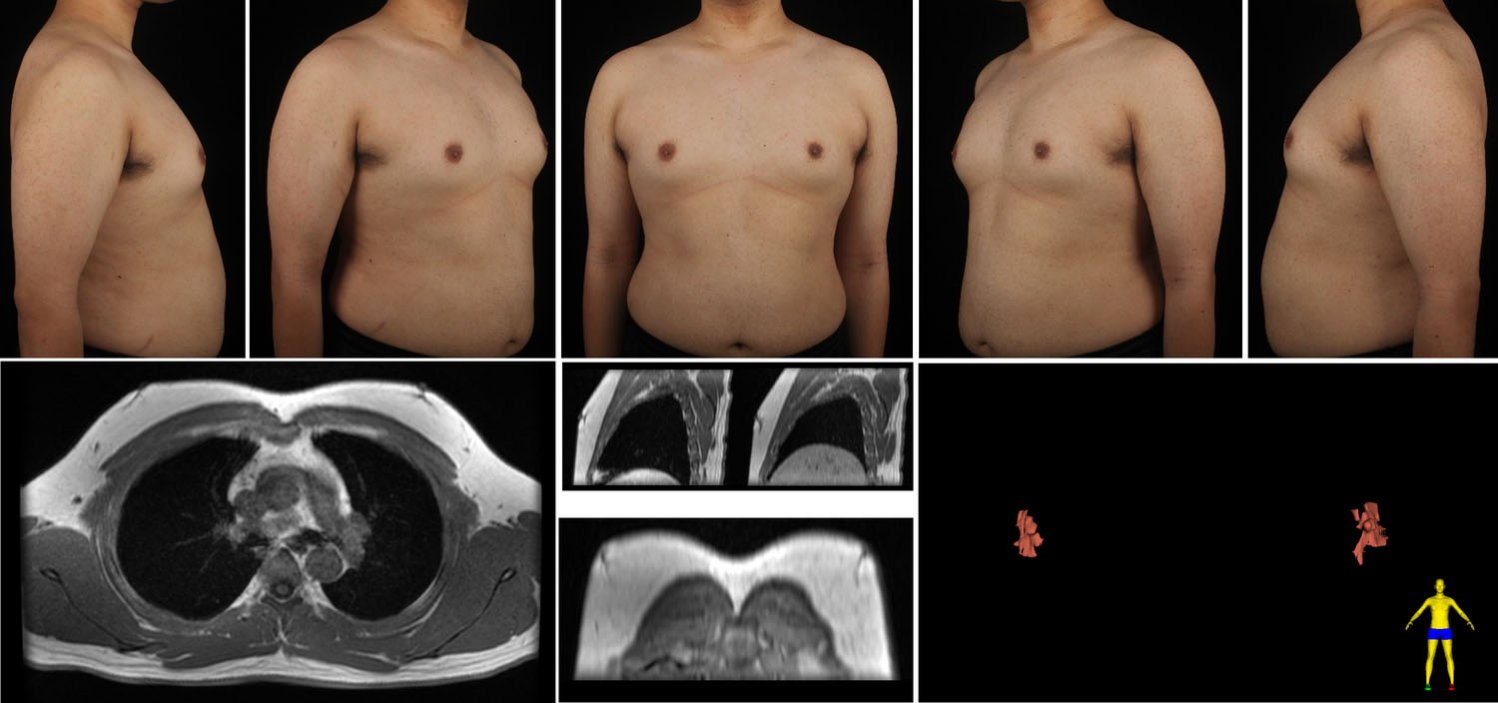
Type 1A gynecomastia. Preoperative chest appearance (upper) and breast MR images (lower) of a 29-year-old man with type 1A gynecomastia

**Fig. 9:**
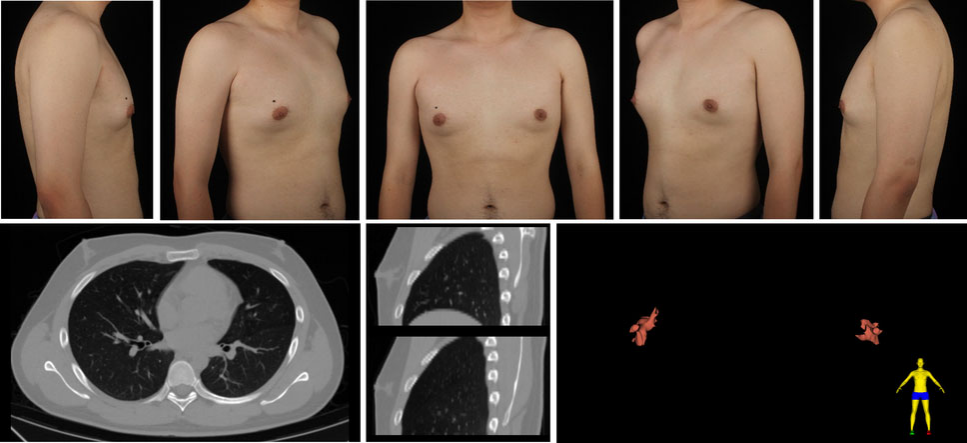
Type 1B gynecomastia. Preoperative chest appearance (upper) and chest CT images (lower) of a 26-year-old man with type 1B gynecomastia

**Fig. 10:**
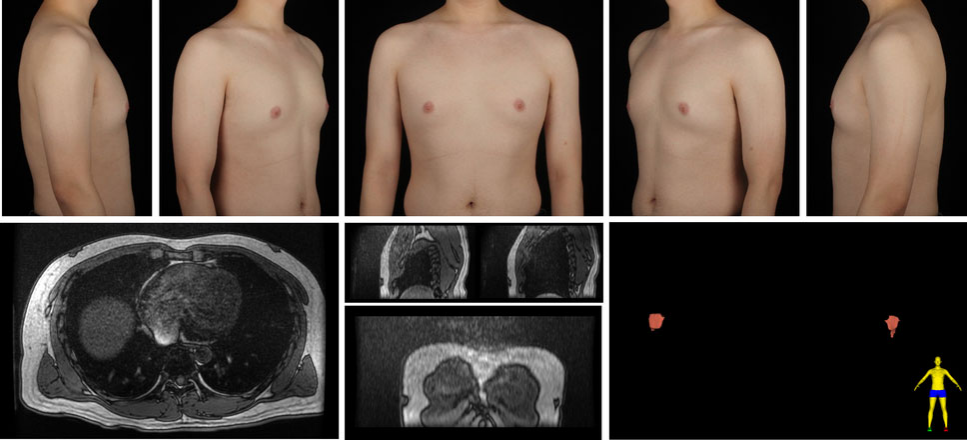
Type 1C gynecomastia. Preoperative chest appearance (upper) and breast MR images (lower) of a 30-year-old man with type 1C gynecomastia

**Fig. 11:**
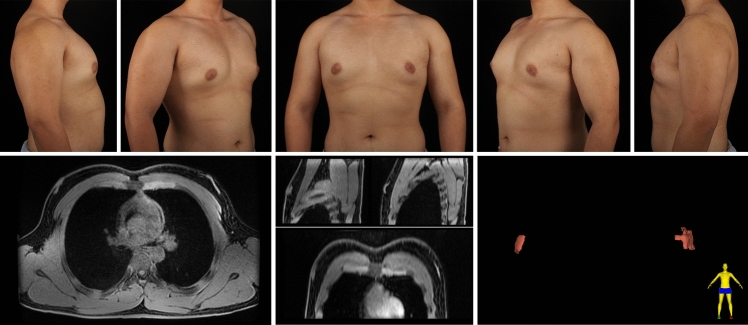
Type 2A gynecomastia. Preoperative chest appearance (upper) and breast MR images (lower) of an 18-year-old man with type 2A gynecomastia

**Fig. 12:**
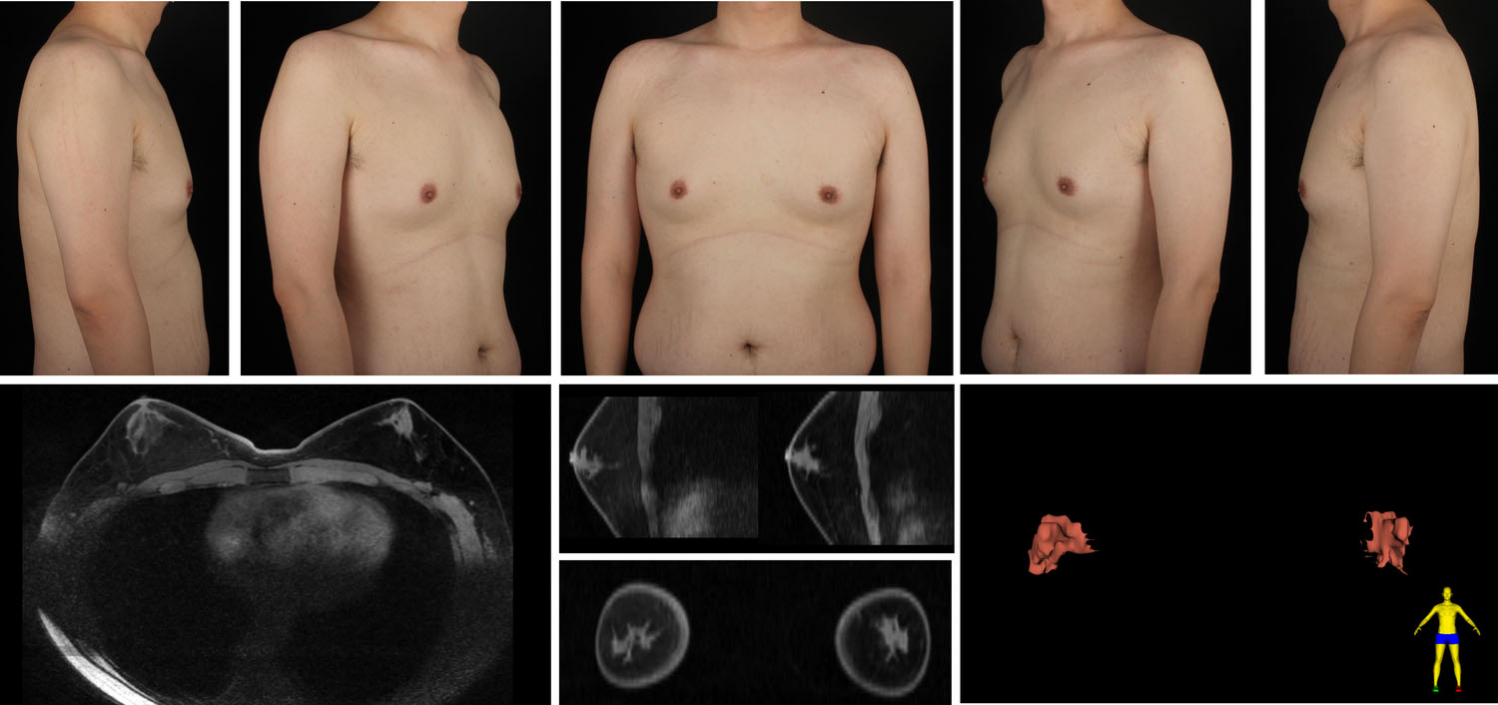
Type 2B gynecomastia. Preoperative chest appearance (upper) and breast MR images (lower) of a 33-year-old man with type 2B gynecomastia

**Fig. 13:**
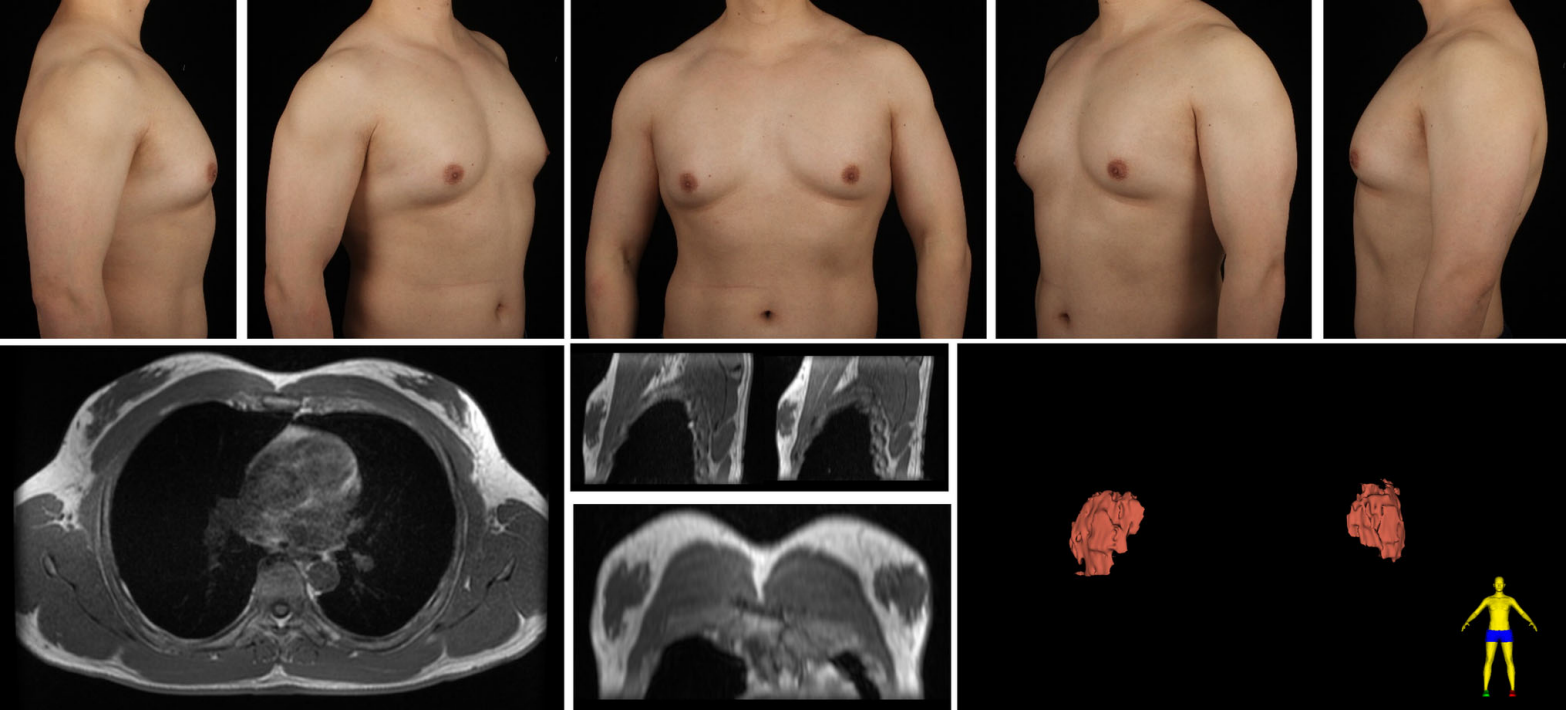
Type 2C gynecomastia. Preoperative chest appearance (upper) and breast MR images (lower) of a 42-year-old man with type 2C gynecomastia

A total of 112 patients underwent MR examinations, while 20 received CT scans. The types of symmetrical gynecomastia and corresponding gland characteristics derived from MR/CT images are shown in Table 4. Figure 8 depicts a typical type 1A gynecomastia MR images. In the transverse plane, the gland tissue is stripe-shaped, while in 3D reconstruction, it appears as a small, flat-patch shape. Figure 9 presents a typical type 1B gynecomastia CT images. The gland appears dendritic in both transverse view and 3D reconstructed images, with the main trunk of the glandular tissue being relatively long and extending deep underneath the NAC. Figure 10 illustrates a typical type 1C gynecomastia MR images. The gland appears as localized small nodules in both transverse plane and 3D reconstructed images. Figure 11 displays a typical type 2A gynecomastia MR images. The gland appearance in both transverse view and 3D reconstructed images resembles that of type 1A, but the distribution area is larger, extending beyond the NAC boundary by 1.5 cm. Figure 12 illustrates a typical type 2B gynecomastia MR images. Both transverse and 3D reconstructed images show a large area with a branched morphology. Figure 13 presents a typical type 2C gynecomastia MR images. The gland appears as large, dense, lump-like masses.

**Table 4:** Type of symmetrical gynecomastia patients

Type	Number of patients	Right gland volume (cm^3^)	Left gland volume (cm^3^)	Right fat tissue volume (cm^3^)	Left fat tissue volume (cm^3^)
Type 1A	20	1.22 ± 0.31	1.12 ± 0.48	232.82 ± 73.31	245.39 ± 89.72
Type 1B	18	2.46 ± 0.99	2.07 ± 0.69	350.48 ± 173.34	363.12 ± 192.38
Type 1C	47	2.07 ± 1.11	2.04 ± 0.87	180.04 ± 86.05	179.89 ± 85.95
Type 2A	13	3.09 ± 0.77	3.61 ± 1.08	223.64 ± 68.65	217.49 ± 67.20
Type 2B	7	6.60 ± 0.76	5.98 ± 0.68	508.35 ± 83.26	529.92 ± 87.67
Type 2C	19	15.68 ± 13.74	15.67 ± 15.01	181.50 ± 134.56	189.64 ± 152.20

Gland and fat tissue volume data are presented as mean ± standard deviation.

### Asymmetric Cases

**Fig. 14:**
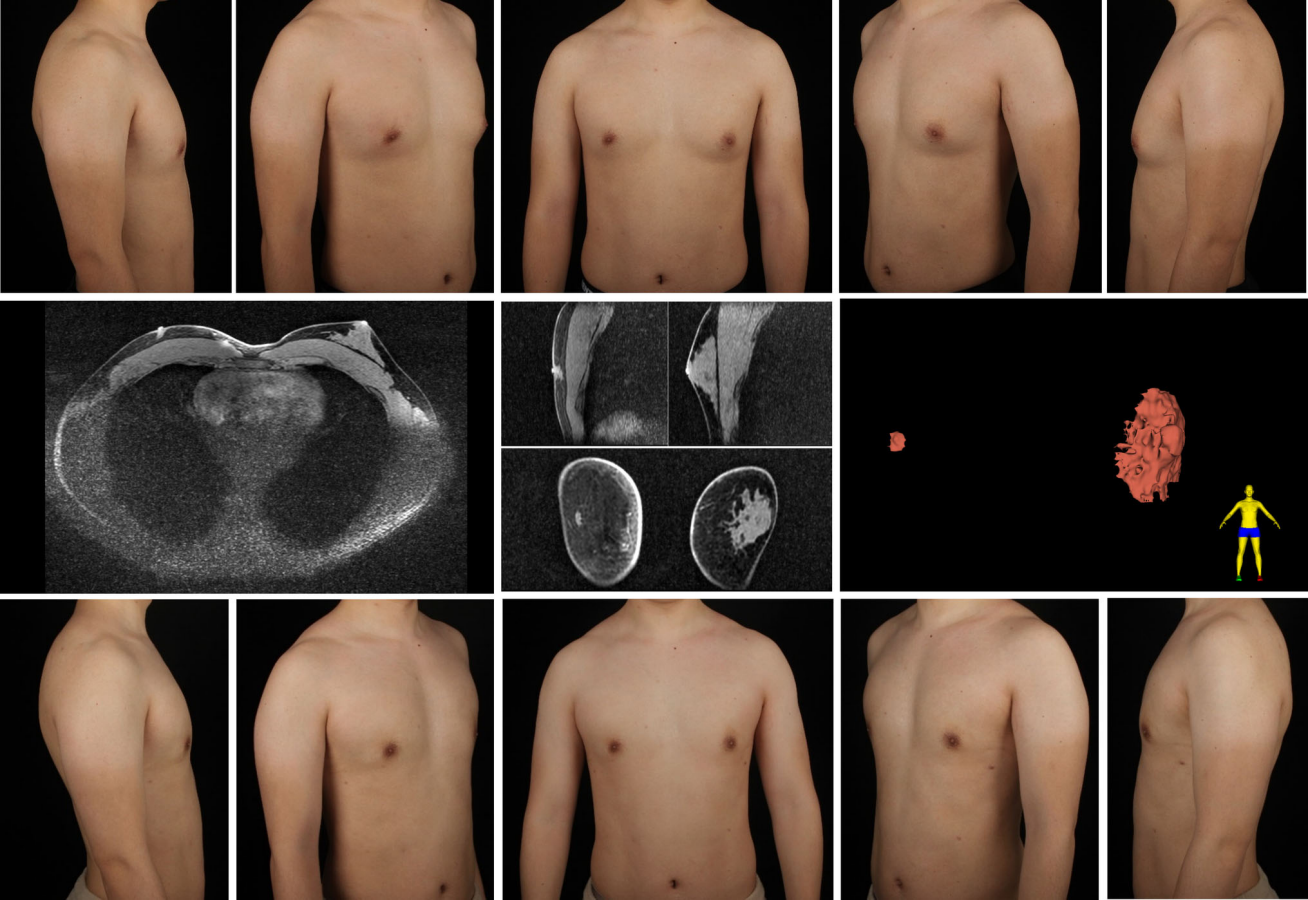
Asymmetric gynecomastia. A 29-year-old man showed grade 1/type 1C (right side) and grade 2/type 2C (left side) gynecomastia, based on chest appearance (upper) and MR images (middle). Liposuction alone was performed on region 1 of right chest, while both mammotome-assisted resection and liposuction were performed on region 1–2 of left chest. Chest appearance 6 months after surgery (lower)

**Fig. 15:**
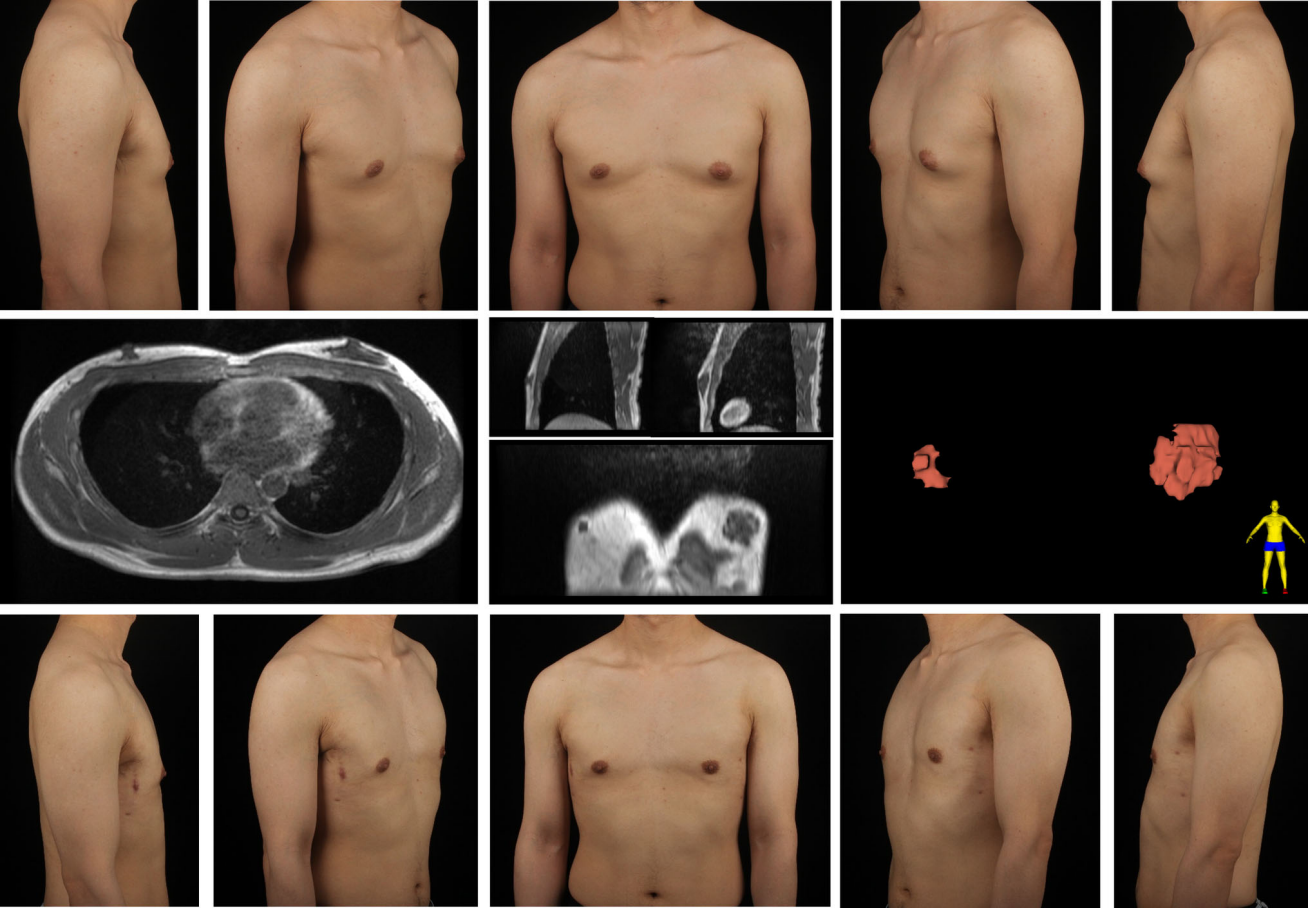
Asymmetric gynecomastia. A 30-year-old man showed grade 1/type 1C (right side) and grade 2/type 2C (left side) gynecomastia, based on chest appearance (upper) and MR images (middle). Liposuction and pull-through were performed on region 1 of right chest, while mammotome-assisted resection and liposuction were performed on region 1–2 of left chest. Chest appearance 6 months after surgery (lower)

There were 20 asymmetric gynecomastia cases in this study. In our classification system, each side is evaluated independently for both grade and type. There is no requirement for the two sides to have the same grade or type classification, and the surgical plan for each side may differ accordingly. Figure 14 depicts a patient exhibiting grade 1 gynecomastia on the right breast and grade 2 on the left. MR images reveal a type 1C gland appearance on the right and type 2C on the left. Liposuction was performed on both sides, while mammotome-assisted resection was also conducted to remove the dense gland tissue in left breast. Figure 15 shows a patient with grade 1 gynecomastia on the right and grade 2 on the left. MR images indicate type 1C (right) and 2C (left) gland appearance. Liposuction and PT were performed on region 1 of right chest, while mammotome-assisted resection and liposuction were performed on region 1–2 of left chest.

## Discussion

Gynecomastia is a benign condition for which surgical treatment has evolved significantly. The requirements for surgery have developed toward minimizing incision size, enhancing postoperative recovery, and improving the chest’s esthetic appearance. Correspondingly, surgical techniques have advanced from initial open incisions around NAC to contemporary minimally invasive procedures [9, 16]. Traditional classification systems, such as those by Simon and Rohrich, often rely on surgeons’ subjective empirical judgment and involve physical examinations that are hard to standardize. This subjectivity may lead to different assessments for the same patient by different surgeons. Given the diversity in patient demographics, chest appearances, and degrees of skin redundancy, crafting standardized and personalized surgical plans is still challenging.

In most classification theory, estimating the proportion of adipose to glandular tissue in the breast is crucial. The removal of soft fat tissue by liposuction and the removal of glandular tissue by direct excision are widely accepted among surgeons. However, most classification systems conflate tissue composition with the degree of breast ptosis or skin redundancy. This confusion complicates the selection of appropriate surgical techniques and the predetermined surgical regions. As a result, these systems may be difficult to provide clear and straightforward guidance for clinical practice [17]. Based on this, we summarized the gynecomastia cases treated at our institution. By analyzing patients’ chest appearances and MR/CT images, we separated the concept grade and type of gynecomastia. This approach enables us to clearly distinguish the choice of surgical techniques and corresponding surgical regions. This classification method provides clearer and more effective guidance for creating personalized surgical plans.

Firstly, the grade of gynecomastia and corresponding surgical region are determined by the appearance of the chest. The degree of breast ptosis and skin redundancy have long been important indicators of gynecomastia classification [18]. The classification methods proposed by Adriana Cordova and Barros are based on the degree of breast ptosis [19, 20]. We add the judgment of skin redundancy in Simon’s classification method and proposed our classification theory. The primary distinction between grade 1 and grade 2 is the presence or absence of IMF. The differentiation between grade 2 and grade 3 hinges on the presence or absence of skin redundancy. Meanwhile, the key difference among grades 3–5 is defined by the positional relationship between the NAC and IMF.

To standardize our approach and improve communication with patients, we have delineated the chest area based on Robert C. Caridi’s theory [21]. Different grades correspond to different surgical regions. The lateral chest and accessory breast areas, which are primarily composed of adipose tissue, are considered relatively independent and are not directly associated with breast ptosis. Thus, the presence of breast ptosis does not necessarily coincide with protrusion in these areas, nor does protrusion in these areas imply severe breast ptosis. Building on this framework, we incorporate Hoyos Alfredo E's insights on definitive chest liposuction to include the lateral chest and accessory breast regions in our classification system [22].

Secondly, the type of gynecomastia and corresponding surgical techniques are determined by the appearance of gland. We defined the boundary for evaluating glandular distribution as 1.5 cm beyond the margins of the NAC. This boundary was proposed based on our clinical experience and corresponds anatomically to region 0 in our chest segmentation framework. Based on previous imaging studies in male breast tissue, we summarized common patterns of gland morphology and distribution, which, together with our defined regional boundaries, inform the choice of surgical technique. Due to the limitation of early imaging techniques, mammography is the main examination method, which has certain sensitivity and accuracy. Appelbaum, A. H. et al. categorized gynecomastia glands into nodular, dendritic, and diffuse types based on their appearance in mammography images. This classification method is classic and still in use today [23]. However, the use of X-rays, which emit a certain level of radioactivity, poses specific limitations. In contrast, ultrasonography is easy to operate and non-radioactive. Nevertheless, its effectiveness heavily depends on the sonographer’s experience. Ultrasonography also tends to have lower sensitivity, and it may not provide a clear and comprehensive visualization of the glandular tissue [24]. Some scholars have proposed classification theory based on ultrasonography results [25]. However, their effectiveness depends heavily on the sonographers’ experience and skill, which may limit their widespread adoption and application. Consequently, mammography and ultrasonography are more commonly used to exclude masses or lesions in the chest’s subcutaneous tissue. Surgeons primarily rely on visual examination and palpation to empirically determine the distribution of glandular tissue in patients. With advances in radiology techniques, MR/CT imaging has increasingly been utilized in the diagnosis and treatment planning of gynecomastia. Zenan Xia et al. evaluated the advantages and disadvantages of MR compared to ultrasonography in the preoperative assessment of gynecomastia. They concluded that MR imaging surpasses ultrasonography in diagnostic accuracy and in providing more precise guidance for surgical interventions [26]. Yakup Cil et al. utilized CT imaging to quantify the volume and ratio of adipose to breast tissue. The ratio was used to evaluate the amount of fat and glandular tissue, thereby helping to determine the appropriate surgical technique [27]. The purpose of this theory is to help choosing the suitable surgical techniques. It gives a more detailed and specific analysis of the adipose and gland tissue. This approach gives certain enlightening meaning.

Mladick Richard A proposed a theory suggesting that all gynecomastia patients could benefit from liposuction combined with varying open incision techniques, depending on the degree of breast ptosis [28]. This theory may not be universally applicable. As mentioned above, patients with only fat tissue or only a small amount of gland tissue beneath NAC can be treated with liposuction alone. In fact, liposuction can effectively remove soft fatty tissue, while dense glandular tissue typically requires excision. If conditions permit, energy-assisted liposuction techniques, such as ultrasonic liposuction and VASER, are also recommended. These methods can more effectively remove fatty tissue, promote skin retraction, and create a more precise and esthetically pleasing body contour. Additionally, we require all surgical patients to wear compression garments for at least 1-month postoperatively. Follow-up observations show that this method effectively promotes skin retraction and tightening, proving to be an economical and effective postoperative recovery measure. When available, devices such as Renuvion or BodyTite are also recommended, which can further enhance skin retraction and tightening. Therefore, using imaging data to determine the composition of chest subcutaneous tissue and to guide the selection of surgical techniques holds significant practical value. Based on the analysis of preoperative MR/CT imaging data from gynecomastia patients, we have identified two primary imaging types and their corresponding surgical techniques. Honestly speaking, although MR/CT imaging offers a reliable modality for preoperative evaluation, these techniques are relatively expensive and time-consuming. In contrast, three-dimensional (3D) ultrasound is more cost-effective and convenient to perform and thus holds promise as an alternative for evaluating mammary gland tissue in gynecomastia patients. If the cost of 3D ultrasound probes can be further reduced and the resolution for detecting mammary gland tissue improved-along with support from well-designed clinical studies—3D ultrasound may become a widely adopted and effective tool for the diagnosis and management of gynecomastia.

## Conclusion

Previous clinical classification methods for gynecomastia have conflated the concepts of grade and type, complicating the selection of appropriate surgical techniques. We propose a clear distinction: the appearance of the chest should determine the grade and corresponding surgical region, while the distribution of mammary gland tissue should define the type and corresponding surgical techniques. This comprehensive and distinct classification theory holds significant application value, offering clearer guidelines for optimizing surgical treatments for gynecomastia.

## Limitation

All cases included in this study were from a single research center, which may introduce some bias. The results would be more robust and reliable if cases from other hospitals were incorporated. This study is a retrospective analysis and may be subject to certain inherent biases. Future prospective studies and external validations are warranted to further refine and confirm the validity of our proposed classification theory.

## References

[1] Billa E, Kanakis GA, Goulis DG. Imaging in gynecomastia. Andrology. 2021;9(5):1444–56. 34033252 10.1111/andr.13051

[2] Narula HS, Carlson HE. Gynaecomastia–pathophysiology, diagnosis and treatment. Nat Rev Endocrinol. 2014;10(11):684–98. 25112235 10.1038/nrendo.2014.139

[3] Daniels J, Brickstock A, Charlton R. Gynaecomastia. BMJ. 2022;379: e069771. 36265883 10.1136/bmj-2021-069771

[4] Webster JP. Mastectomy for gynecomastia through a semicircular intra-areolar incision. Ann Surg. 1946;124:557–75. 20997808

[5] Simon BE, Hoffman S, Kahn S. Classification and surgical correction of gynecomastia. Plast Reconstr Surg. 1973;51(1):48–52. 4687568 10.1097/00006534-197301000-00009

[6] Rohrich RJ, Ha RY, Kenkel JM, et al. Classification and management of gynecomastia: defining the role of ultrasound-assisted liposuction. Plast Reconstr Surg. 2003. 10.1097/01.PRS.0000042146.40379.25. 12560721 10.1097/01.PRS.0000042146.40379.25

[7] Prasetyono TOH, Andromeda I, Budhipramono AG. Approach to gynecomastia and pseudogynecomastia surgical techniques and its outcome: a systematic review. J Plast Reconstr Aesthet Surg. 2022;75(5):1704–28. 35304857 10.1016/j.bjps.2022.02.008

[8] Holzmer SW, Lewis PG, Landau MJ, et al. Surgical management of gynecomastia: a comprehensive review of the literature. Plast Reconstr Surg. 2020;8(10): e3161. 10.1097/GOX.0000000000003161 PMC7647635 33173677

[9] Wang Y, Wang J, Liu L, et al. Comparison of curative effects between mammotome-assisted minimally invasive resection (MAMIR) and traditional open surgery for gynecomastia in Chinese patients: a prospective clinical study. Breast J. 2019;25(6):1084–9. 31267613 10.1111/tbj.13424

[10] Fruhstorfer BH, Malata CM. A systematic approach to the surgical treatment of gynaecomastia. Br J Plast Surg. 2003;56(3):237–46. 12859919 10.1016/s0007-1226(03)00111-5

[11] Monarca C, Rizzo MI. Gynecomastia: tips and tricks-classification and surgical approach. Plast Reconstr Surg. 2013;131(5):863e-e865. 23629140 10.1097/PRS.0b013e318287a18f

[12] Cardenas-Camarena L, Dorado C, Guerrero MT, et al. Surgical masculinization of the breast: clinical classification and surgical procedures. Aesthet Plast Surg. 2017;41(3):507–16. 10.1007/s00266-016-0731-9 28341946

[13] Innocenti A, Melita D, Innocenti M. Gynecomastia and chest masculinization: an updated comprehensive reconstructive algorithm. Aesthet Plast Surg. 2021;45(5):2118–26. 10.1007/s00266-021-02275-7 33939025

[14] Morselli PG. Pull-through: a new technique for breast reduction in gynecomastia. Plast Reconstr Surg. 1996;97(2):450–4. 8559832 10.1097/00006534-199602000-00028

[15] Hammond DC, Arnold JF, Simon AM, et al. Combined use of ultrasonic liposuction with the pull-through technique for the treatment of gynecomastia. Plast Reconstr Surg. 2003. 10.1097/01.PRS.0000072254.75067.F7. 12960873 10.1097/01.PRS.0000072254.75067.F7

[16] Varlet F, Esposito C, Scalabre A, et al. Pediatric endoscopic subcutaneous mastectomy (pesma) with liposuction in adolescents with gynecomastia. Surg Endosc. 2023;37(1):766–73. 36050608 10.1007/s00464-022-09550-x PMC9839820

[17] Waltho D, Hatchell A, Thoma A. Gynecomastia classification for surgical management: a systematic review and novel classification system. Plast Reconstr Surg. 2017;139(3):638e-e648. 28234829 10.1097/PRS.0000000000003059

[18] Ratnam BV. A new classification and treatment protocol for gynecomastia. Aesthet Surg J. 2009;29(1):26–31. 19233002 10.1016/j.asj.2008.11.003

[19] Cordova A, Moschella F. Algorithm for clinical evaluation and surgical treatment of gynaecomastia. J Plast Reconstr Aesthet Surg. 2008;61(1):41–9. 17983883 10.1016/j.bjps.2007.09.033

[20] Barros ACSDD, Sampaio MDCM. Gynecomastia: physiopathology, evaluation and treatment. Sao Paulo Med J. 2012;130(3):187–97. 22790552 10.1590/S1516-31802012000300009 PMC10876201

[21] Caridi RC. Defining the aesthetic units of the male chest and how they relate to gynecomastia based on 635 patients. Plast Reconstr Surg. 2018;142(4):904–7. 30252811 10.1097/PRS.0000000000004807

[22] Hoyos AE, Perez ME, Domínguez-Millán R. Gynecomastia treatment through open resection and pectoral high-definition liposculpture. Plast Reconstr Surg. 2021;147(5):1072–83. 33890890 10.1097/PRS.0000000000007901

[23] Mannix J, Duke H, Almajnooni A, et al. Imaging the male breast: gynecomastia, male breast cancer, and beyond. Radiographics. 2024;44(6): e230181. 38752766 10.1148/rg.230181

[24] Sarıca Ö, Kahraman AN, Öztürk E, et al. Efficiency of imaging modalities in male breast disease: can ultrasound give additional information for assessment of gynecomastia evolution? Eur J Breast Health. 2018;14(1):29–34. 29322116 10.5152/ejbh.2017.3416 PMC5758060

[25] Klinger M, Bandi V, Giannasi S, et al. Gynecomastia: ultrasound-confirmed classification pertainent to surgical correction. Aesthet Plast Surg. 2021;45(4):1397–403. 10.1007/s00266-021-02187-6 33625529

[26] Xia Z, Ding N, Kang Y, et al. Is breast magnetic resonance imaging superior to sonography in gynecomastia evaluation and surgery planning. Aesthet Plast Surg. 2023;47(5):1759–70. 10.1007/s00266-023-03506-9 37500904

[27] Cil Y, Alparslan MB, Aktas G, et al. Adipose tissue measurement in gynecomastia with computerized tomography. Erciyes Tıp Dergisi/Erciyes Med J. 2012;34(1):15–9.

[28] Mladick RA. Body contouring: gynecomastia. Aesthet Surg J. 2004;24(5):471–9. 19336198 10.1016/j.asj.2004.06.005

